# Deep Learning Models to Reduce Stray Light in TJ-II Thomson Scattering Diagnostic

**DOI:** 10.3390/s24092764

**Published:** 2024-04-26

**Authors:** Ricardo Correa, Gonzalo Farias, Ernesto Fabregas, Sebastián Dormido-Canto, Ignacio Pastor, Jesus Vega

**Affiliations:** 1 Escuela de Ingeniería Eléctrica, Pontificia Universidad Católica de Valparaiso, Av. Brasil 2147, Valparaiso 2362804, Chile; ricardo.correa.t@mail.pucv.cl (R.C.); gonzalo.farias@pucv.cl (G.F.); 2 Departamento de Informática y Automática, Universidad Nacional de Educación a Distancia (UNED), Juan del Rosal 16, 28040 Madrid, Spain; efabregas@dia.uned.es; 3 Laboratorio Nacional de Fusión, CIEMAT, Avda. Complutense 40, 28040 Madrid, Spain; ignacio.pastor@ciemat.es (I.P.);

**Keywords:** generative adversarial network, nuclear fusion energy, stray light

## Abstract

Nuclear fusion is a potential source of energy that could supply the growing needs of the world population for millions of years. Several experimental thermonuclear fusion devices try to understand and control the nuclear fusion process. A very interesting diagnostic called Thomson scattering (TS) is performed in the Spanish fusion device TJ-II. This diagnostic takes images to measure the temperature and density profiles of the plasma, which is heated to very high temperatures to produce fusion plasma. Each image captures spectra of laser light scattered by the plasma under different conditions. Unfortunately, some images are corrupted by noise called stray light that affects the measurement of the profiles. In this work, we propose the use of deep learning models to reduce the stray light that appears in the diagnostic. The proposed approach utilizes a Pix2Pix neural network, which is an image-to-image translation based on a generative adversarial network (GAN). This network learns to translateimages affected by stray light to images without stray light. This allows for the effective removal of the noise that affects the measurements of the TS diagnostic, avoiding the need for manual image processing adjustments. The proposed method shows a better performance, reducing the noise up to 98% inimages, which surpassesprevious works that obtained 85% for the validation dataset.

## 1. Introduction

Nuclear fusion is the process that powers the stars, such as our sun. Potentially, this energy source could supply all the needs of the world for millions of years [1]. In contrast to fission, the process releases large amounts of energy when a couple of atomic nuclei fuse in a heavier nucleus. The fusion community has made a great effort to make possible fusion energy. Nowadays, there are many experimental fusion devices in operation to control this highly complex process. To this end, several experiments (called discharges or shots) are performed to understand the physics of plasma, which is an ionized gas that is magnetically confined inside the thermonuclear devices [2].

During the discharges, many data acquisition systems around the device acquire data at a very high sampling frequency, which generates massive databases per campaign. Bolometry, density, temperature, and soft X-rays are just some examples of the thousands of data acquired during a discharge. Huge databases, with an enormous amount of data, are a common situation in experimental fusion devices [3].

It is important to emphasize the use of machine learning techniques in fusion devices for the real-time recognition of events. In particular, these techniques have been shown to be effective in the prediction of incoming disruptions [4]. Disruptions are produced by sudden plasma instabilities that provoke a rapid loss of confinement (which occurs in specific fusion devices called tokamaks). As a result, disruptions can damage the surrounding plasma structures irreversibly. Therefore, their prediction in advance is essential for some new tokamak devices (Japan Torus 60 Super Advanced (JT-60SA), located in Naka, Ibaraki, Japan [5], International Thermonuclear Experimental Reactor (ITER), located in Saint-Paul-lez-Durance, France [6,7], or the DEMOnstration power plant (DEMO), which location is not defined yet [8]) to trigger avoidance actions or, in the case of imminent disruption, to fire mitigation remedies.

Due to the high cost of collecting new data in nuclear fusion experiments, it is crucial to find ways to access additional discharges or experimental runs. Therefore, it is highly desirable to explore creating probabilistic models to generate new synthetic, but realistic, fusion data. These models learn from existing datasets, capturing the underlying relationships between the measured signals.

The potential use of generative deep learning models for this purpose was previously explored in [9]. In this article, we move forward from that work to validate the application and usefulness of such deep learning generative models on the existing nuclear fusion databases. Particularly, a type of generative adversarial network (GAN) was applied for noise reduction on the Thomson scattering (TS) diagnostic of the TJ-II experimental fusion device. The Thomson scattering diagnostic measures density and temperature profiles by processing five types of images [10,11]. Each image captures spectra of laser light scattered by the plasma.

Four types of such TS images are frequently corrupted by noise called stray light. The noise normally appears on the left side of captured images. This disturbance could produce unreliable measurements of the temperature and density profiles. That is why it is required that one applies a noise reduction technique to the acquired TS images before the reconstruction of the profiles. Note that the stray light can be mixed with significant information captured for the image, and therefore, the noise reduction can affect further measurements. There are some classical solutions (e.g., notch laser-line rejection filters) that could be applied to reduce the noise; however, since the shape of the noise is not regular, the results with these solutions are not suitable. On the contrary, some advanced image processing algorithms have shown very good results when trying to reduce the noise without affecting most of the acquired data [12,13].

Although the performance of such advanced algorithms has been very satisfactory, these techniques always need a manual tuning of some important parameters. For that reason, the study of new approaches is needed that allow avoiding, or at least minimizing, such manual settings of the algorithms to reduce the disturbance of stray light.

Our approach proposes the training of a deep neural network to perform the task of mapping one TS image from the original domain (TS images with noise) into another domain (TS images without noise). Specifically, a GAN was implemented to reduce noise in the TS diagnostic of the TJ-II experimental fusion device. By utilizing a simple GAN with nuclear fusion data, new synthetic data are probabilistically generated that closely resemble data from a nuclear fusion experiment. However, these new data still exhibited the same issue of stray light as the original data, so a variant of this network known as pix2pix GAN was implemented. This work describes the application of the pix2pix GAN for noise reduction and shows a comparison with previous work to decrease the effect of stray light from TS images.

The main contributions of this paper are the following:

- Application of deep learning models to generate realistic data and images from nuclear fusion databases.
- Application of GANs for stray light (noise) removal in Thomson scattering diagnostic images.
- Reduction by the proposed modelof the manual selection of tuning of the main parameters. It does not use design parameters to obtain similar or better results than those obtained by previous methods.
- Comparison of the proposed model with techniques developed in previous works [12,13].

The rest of this paper is structured as follows. Section 2 introduces some basic aspects of nuclear fusion energy and depicts the images of the Thomson scattering diagnostic. Section 3 describes the basis of the GAN models. Section 4 presents the developed algorithm to eliminate the noise of nuclear fusion images. Section 5 presents and analyzes the obtained results of this work. Finally, the main conclusion and future works are presented.

## 2. Background

### 2.1. Nuclear Fusion

In nuclear fusion devices, the plasma (gaslike state of matter with ionized particles) is heated to very high temperatures, around 150 million degrees Celsius. The reactor uses deuterium and tritium [14] to produce the plasma, and magnetic fields are used to confine the plasma inside the chamber. Stellarator [15] and tokamaks [16] are the most common configurations of these devices for the magnetic confinement of the plasma. The fusion process releases energy that could be used to heat water and then powers a turbine generator that produces electricity [17,18].

There are several experimental nuclear fusion devices around the world. The most recent example is the ITER [6,7], which is under construction in France, and it will be the world’s largest and most advanced experimental tokamak nuclear fusion reactor. It aims to be the first to achieve net energy gain (producing more energy than used). ITER is located in Cadarache (France). After ITER, the first commercial demonstration fusion power plant, named DEMO, will be intended.

**Figure 1:**
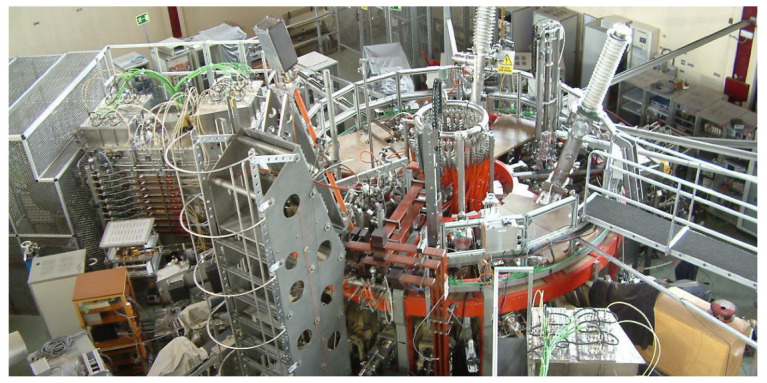
The TJ-II device.

Other notable devices include the following:

- JET located in Culham Centre for Fusion Energy in Oxfordshire, UK is currently the largest operational tokamak reactor [19].
- DIII-D located in San Diego, California, USA, is a tokamak machine developed by General Atomics [20].
- TJ-II located in Madrid, Spain, a medium-sized stellarator for plasma creation and heating [21]. Figure 1 shows a view of the TJ-II device [22].
- The Wendelstein 7-X (W7-X) [23], an experimental stellarator built in Greifswald, Germany, by the Max Planck Institute for Plasma Physics (IPP).
- K-STAR (Korea Superconducting Tokamak Advanced Research) [24], which is at the Korea Institute of Fusion Energy in Daejeon, South Korea.
- Axially Symmetric Divertor Experiment (ASDEX-Upgrade tokamak) [25], is located in Garching, Germany.

### 2.2. Thomson Scattering Diagnostic and Stray Light in TJ-II

**Figure 2:**
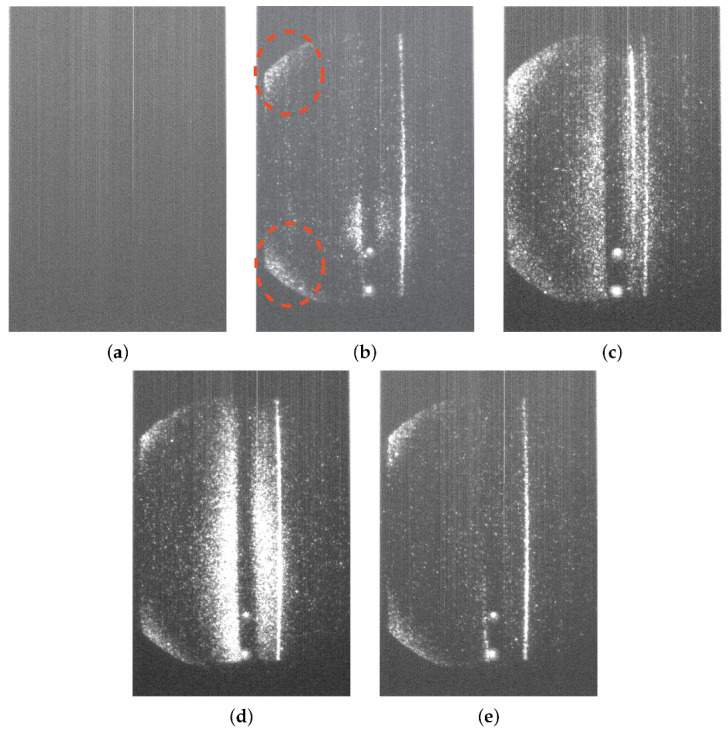
Thomson scattering diagnostic images with noise. The images shown here correspond to preprocessed versions to accentuate the stray light: (**a**) BKG, (**b**) COFF, (**c**) ECH, (**d**) NBI, (**e**) STR. The red circle shows the stray light of a COFF image.

The Thomson scattering (TS) diagnostic system, used to measure plasma properties, is sensitive to stray light. The stray light can contaminate the five different types of images the diagnostic acquires. These images are spectra (color breakdowns) of laser light scattered by the plasma, taken under various conditions. Table 1 and Figure 2 describe the five types of TS images.

In Thomson scattering (TS) diagnostics, stray light emerges as the primary source of noise. This persistent phenomenon has been an ongoing challenge in optical design for such diagnostics [26]. Commonly observed in gas discharges near glass walls, it is caused by various phenomena such as Fresnel reflection on lens surfaces, air bubbles in glass, dust, and diffraction at aperture edges. Additionally, it arises from the scattering of part of the laser light in the static plasma environment and is often much more intense than Thomson scattering itself. Its constant presence degrades both image contrast and measurement accuracy, emphasizing the crucial importance of its control in optimizing the diagnostics.

According to experts in the field, this interference is commonly evident on the left side of images, as highlighted in Figure 2. Without the application of noise reduction techniques, raw images captured by the Charge-Coupled Device (CCD) camera in Thomson scattering diagnostics, often affected by this type of interference, can generate temperature and density profiles lacking reliability for the diagnosis. Additionally, stray light tends to integrate with a substantial portion of relevant information, located in the center of these images, occasionally complicating the distinction between data and noise. Although conventional solutions, such as notch filters in front of the spectrograph [27,28], have been implemented to mitigate stray light, the results are unsatisfactory due to the lack of a predefined location for the noise and its irregular shape. Consequently, both parasitic light and information are equally reduced.

**Figure 3:**
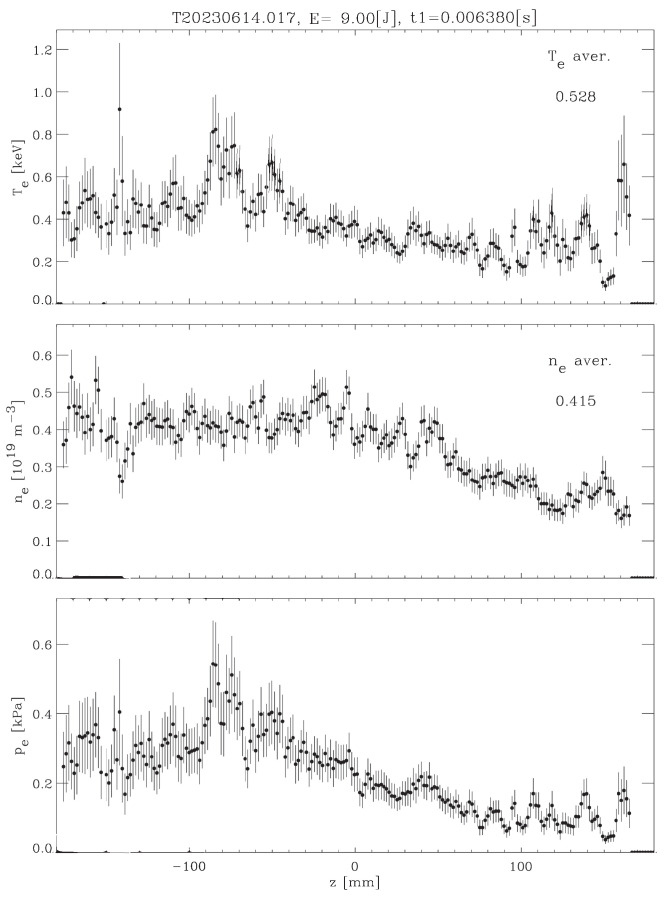
Example of a profile reconstruction.

Figure 3 depicts an example of the Thomson scattering profiles of plasma, where the x axis stands for the position of a small plasma volume along the ruby laser chord traversing the plasma. Hence, each z value labels a point in the plasma column from which the analysis of Thomson scattered light produces the local electron temperature and density.

Uncorrected stray light has two main deleterious effects on Thomson scattering profile reconstruction: on the one hand, stray light contributes additional counts to genuine Thomson scattering ones, to artificially increase, i.e., overestimate, plasma density. On the other hand, stray light tends to broaden the scattering spectrum (overestimating plasma temperature) because it contributes counts overlapping with Thomson scattering in regions of the CCD that would have nearly none of them for a given plasma temperature.

## 3. Generative Modeling

### 3.1. Discriminative Model vs. Generative Model

**Figure 4:**
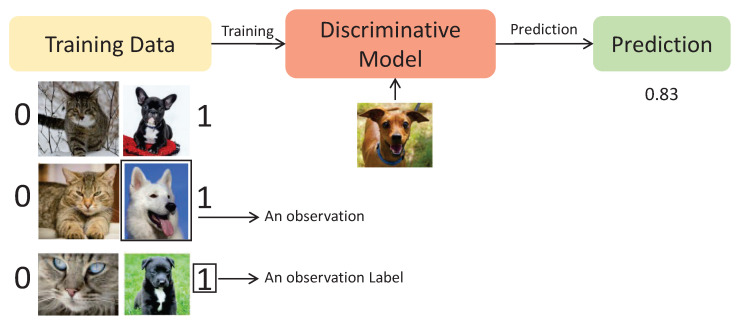
Discriminative model example.

Roughly, a discriminative model, as its name suggests, is a supervised learning black-box model which is trained with labeled data. The model is capable of learning to classify (discriminate) a new observation with a level of precision. Figure 4 shows an example of a discriminative model classifying if a picture is a dog or not [29].

As can be seen, the training dataset contains pictures of dogs labeled with 1 (right side) and pictures of cats labeled with 0 (left side). After the training stage, the model is capable of classifying whether a new picture is a dog or not with a level of precision, in this example 83%. Note that a discriminative model is only capable of classifying a picture, but it cannot generate a new one.

**Figure 5:**
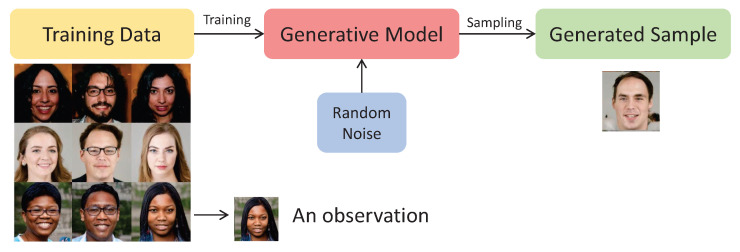
Generative model example.

On the other hand, a generative model has the ability to create a new image based on the training set without the need for labels (unsupervised learning). This model generates fresh and unique instances that closely resemble those present in the original training set. When considering a data set of observations, it is assumed that they are generated according to an undisclosed probabilistic distribution. The goal of a generative model is to emulate this unknown distribution. Success is achieved when the model can produce examples that have characteristics similar to the same unknown distribution observed in the training data set [29]. Figure 5 shows an example of a generative model which is capable of creating a new human face picture from the training data set.

### 3.2. Generative Adversarial Network

A generative adversarial network (GAN) is a machine learning architecture in which two neural networks engage in a competitive process to enhance the accuracy of their predictions. GANs typically operate within the context of unsupervised learning, using a zero-sum cooperative game framework for learning purposes. The two neural networks involved in a GAN setup are called the generator and the discriminator. The generator and discriminator functions in a GAN, respectively, operate as a convolutional and a deconvolutional neural network. The generator’s primary objective is to create outputs that closely resemble genuine data, making them indistinguishable from real data. The discriminator, on the other hand, distinguishes between genuine and artificially generated outputs. GANs can generate their training data. As the iterative process between the adversarial networks continues, the generator gradually improves its ability to produce higher-quality outputs, while the discriminator enhances its proficiency in identifying artificially generated data [30].

**Figure 6:**
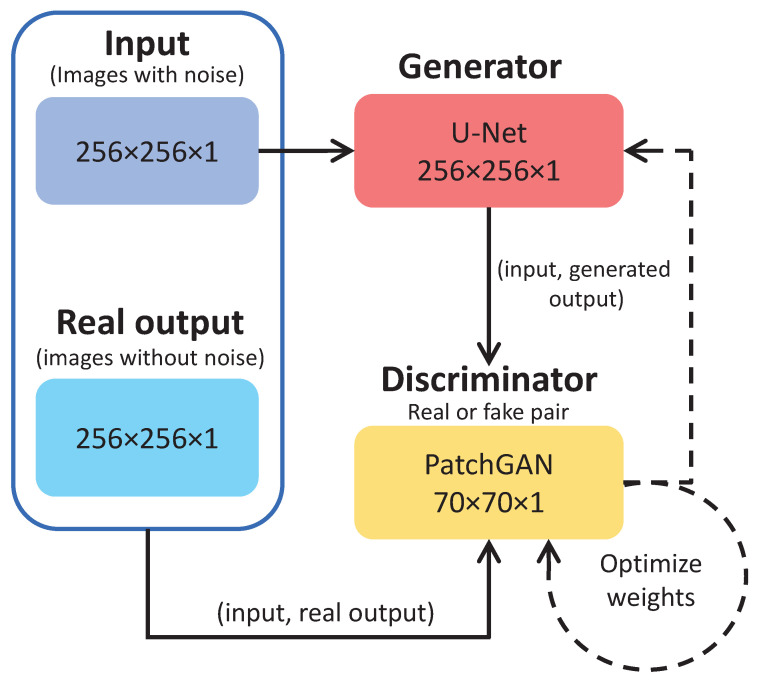
Block diagram of the Pix2Pix GAN.

The conditional generative adversarial network (cGAN) extends the GAN framework by providing precise control over the generated image, such as generating an image of a particular class. A notable implementation of cGAN is the Pix2Pix GAN, which allows conditional image generation based on a given image, making it a versatile method for image-to-image translation tasks. To effectively train the Pix2Pix GAN, data sets must consist of input images (pretranslation) and corresponding output or target images (post-translation). The GAN architecture shown in Figure 6 requires careful configuration of a generator model, a discriminator model, and an optimization procedure. Both the generator and discriminator models use standard convolution-batch-normalization-ReLU layer blocks, consistent with typical deep convolutional neural networks.

## 4. Proposed Approach

Our approach proposes the training of a deep neural network to perform the task of translating one image into another to eliminate the stray light found in the Thomson scattering diagnostic images. This type of network is better known as Pix2Pix GAN and is a type of conditional GAN, where the generation of the output image is conditioned by an input image (target), which is what the initial image (source) is to be translated into. In our case, an image with noise is processed to translate into an image without noise. These images without noise were obtained through an algorithm based on the extraction of regions with connected components (ERCC) [13], which is the one with which we want to compare the efficiency of the noise reduction.

The ERCC algorithm is based on segmentation theory. Segmentation refers to the process of partitioning a digital image into multiple segments. Image segmentation is the process of assigning the same label to a pixel that shares similar visual characteristics. The segmentation partitions an image into connected subimages (regions) such that all regions are disjointed, and the union of all of them makes up the original image.

ERCC can be easily applied to detect the stray light (noise) in the TS images, but although the performance of the ERCC algorithm is very high, it requires a suitable configuration to work successfully. For instance, in [13], it is noted that step 1 (convert the image to a binary image) and step 4 (removing a region according to a criterion) of the ERCC algorithm have an important impact on the whole noise reduction process. The selection of such correct parameters in these steps is crucial for the performance of the ERCC algorithm. That is why an automatic way to achieve stray light reduction, such as a GAN network, motivates this work.

**Figure 7:**
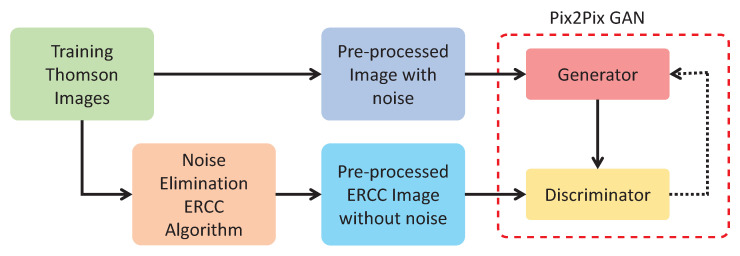
Training GAN block diagram.

To implement the pix2pix GAN (GAN, henceforth, for short) approach, we have a total of 942 images that are randomly divided into 701 for training and 241 for testing. To carry out the model training process, it is necessary to have two sets of data. Set A contains the original images with background stray light, while set B contains the same images as set A, but they are processed with the ERCC algorithm to reduce the stray light. Thus, both sets of training images are input to the GAN network, with set A being the source images of the system and set B being the target images. Note that both sets (A and B) of training images are distributed as follows: BKG (102 images), CUT (85 images), ECH (321 images), NBI (107 images), and STR (86 images). The training images were resized from 385×576 pixels to 448×576 pixels to optimize the algorithm performance and training. Figure 7 shows the block diagram of the proposed approach’s training.

The training process took approximately one week (in a Xeon server with 8 GPUs RTX 2080Ti, from Santa Clara, California, USA), resulting in the successful acquisition of a model. This model was then tested with different images and subsequently validated through an iterative process. As mentioned earlier, the test set comprises 241 images distributed as follows: BKG (50 images), CUT (42 images), ECH (49 images), NBI (50 images), and STR (50 images). To use the model, it is fed a test image, and the model should output the same input image but without the stray light noise (image translation). To evaluate how well the noise of the images is reduced, the mathematical function described in Equation (1) was selected.

(1) $$D\left(I\right)=\left\{\begin{array}{cc} 1, & \delta \mu_{BKG}-3\sigma \mu_{BKG}<\delta \mu_{f}<\delta \mu_{BKG}+3\sigma \mu_{BKG}\\ 0, & otherwise \end{array}\right.$$

**Figure 8:**
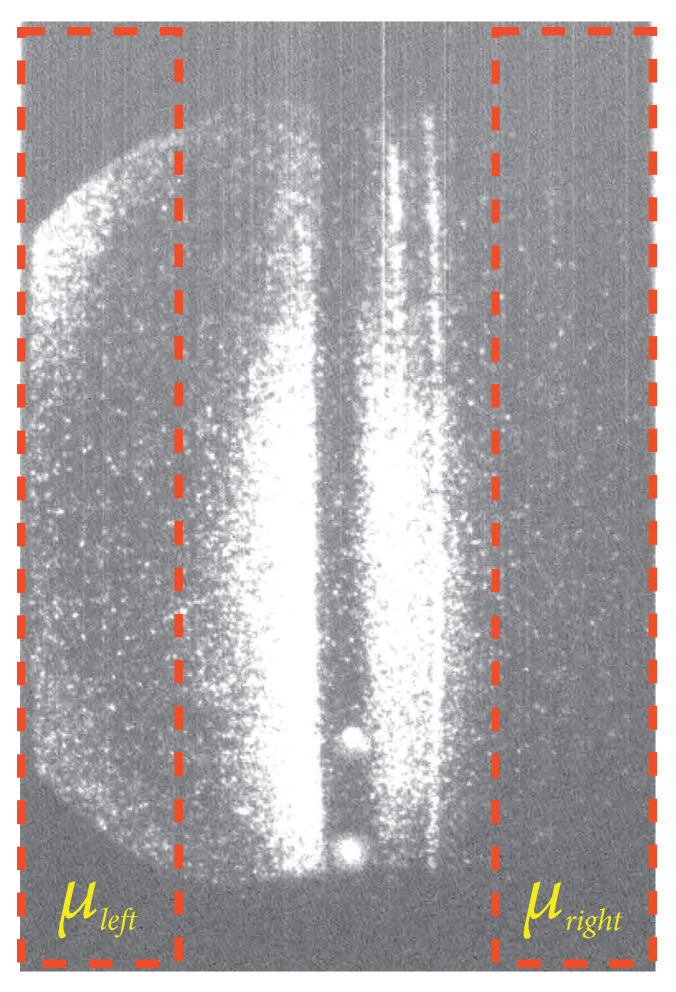
Example of the left and right sides of a TS image to be considered in the validation process.

This function called the denoising function D(I), compares the right and left sides of each image (I), as shown in Figure 8. $\mu_{left}$ and $\mu_{right}$ denote the pixel mean on the left and right sides of each image, respectively. The difference between these means is defined as $\delta \mu_{f}=\mu_{left}-\mu_{right}$. Such a difference is compared with the background images through the computation of $\delta \mu_{BKG}$ and $\sigma \mu_{BKG}$, which represent the mean and standard deviation, respectively, of $\delta \mu_{f}$ for all background images (BKG).

The function D(I) outputs “1” when noise reduction is achieved and 0 otherwise [12]. It is important to note that this function operates under the assumption that the left and right ends of the image, after noise reduction, should be similar. Figure 8 illustrates the division of the left and right ends in a TS image.

**Figure 9:**
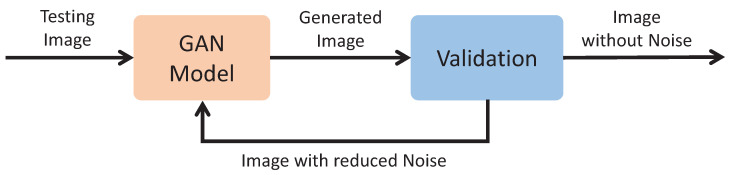
Block diagram of the generation and validation of denoising images.

The application of the proposed process is quite simple, but the noise is not alwaysremoved in one iteration. That is why an iterative version of the algorithm for image generation and validation is introduced. Figure 9 shows the block diagram of this step. As can be seen, the operation of this algorithm follows the same basics of the process explained above, where an image is generated by the GAN model from a test image. After that, the result of the noise reduction is validated by the denoising function, which returns “1” if the noise has been removed, and so the process ends. If the result of the denoising function outputs “0”, it means that there is still noise, and therefore this generated image is introduced again to the model to generate a new image with less noise than the previous step. This process is repeated until the image noise is completely removed or until a maximum number of iterations is reached.

## 5. Experimental Results

Following the successful execution of the training and removal of stray light processes in the images, highly promising results were achieved through the proposed approach. These outcomes demonstrate significant improvements in various aspects compared to previous methods, which will be described in what follows.

**Figure 10:**
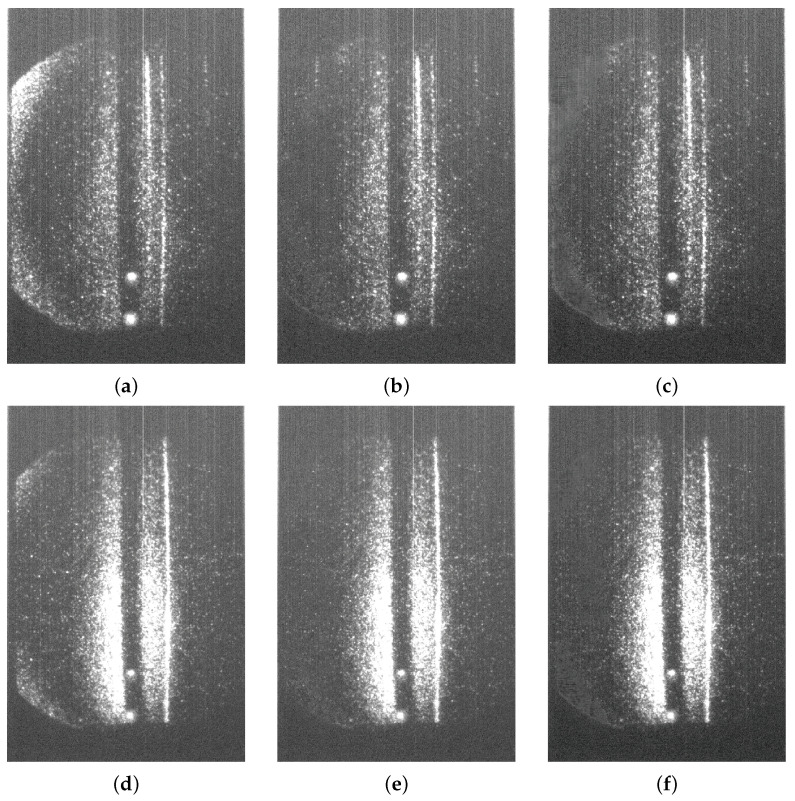
Two examples of noise reduction applications with ERCC and GAN approaches: (**a**) Original ECH, (**b**) ERCC ECH, (**c**) GAN ECH, (**d**) Original NBI, (**e**) ERCC NBI, and (**f**) GAN NBI.

Figure 10 shows a comparative analysis by applying the ERCC and GAN approaches for two TS images. The first row (a, b, and c) illustrates the outcomes for an ECH image, while the second row (d, e, and f) showcases the results for an NBI image. Similar to previous instances, each row displays, from left to right, the original testing image, the noise elimination outcome from the ERCC algorithm, and the GAN denoising generated image.

**Figure 11:**
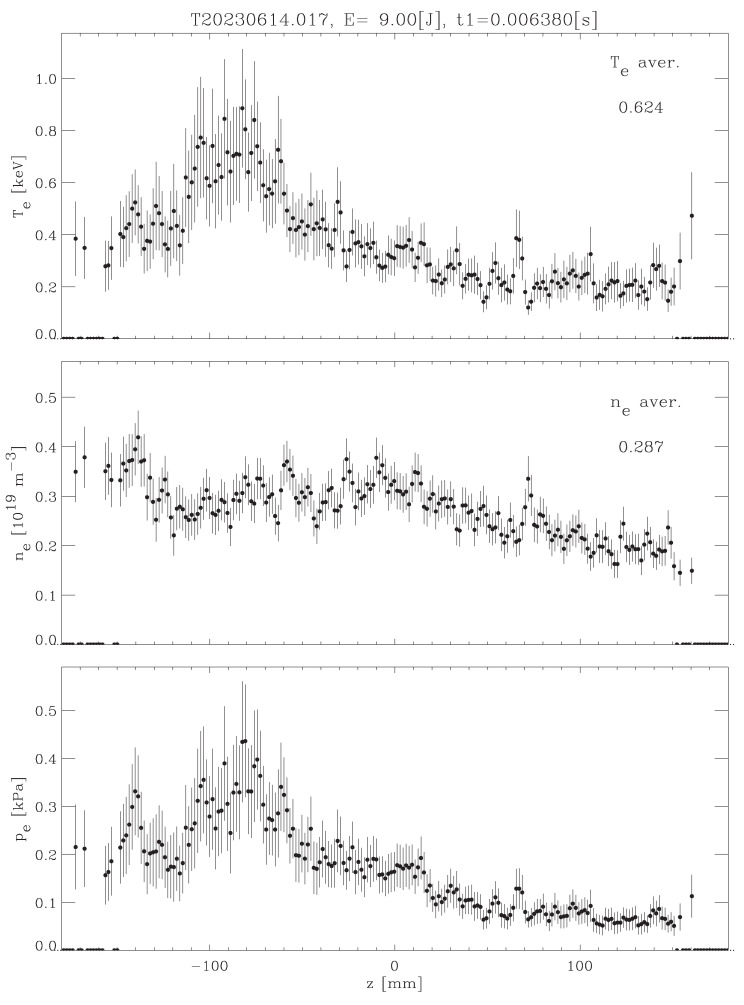
Original profiles of Thomson scattering diagnosis images.

**Figure 12:**
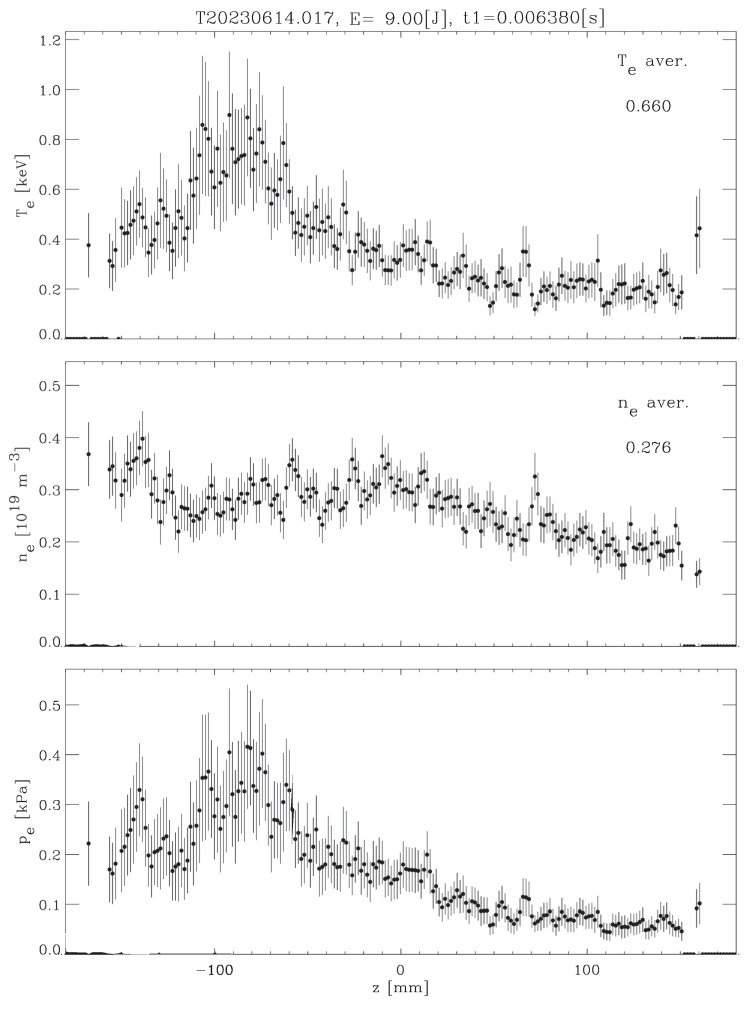
ERCC profiles of Thomson scattering diagnosis images.

**Figure 13:**
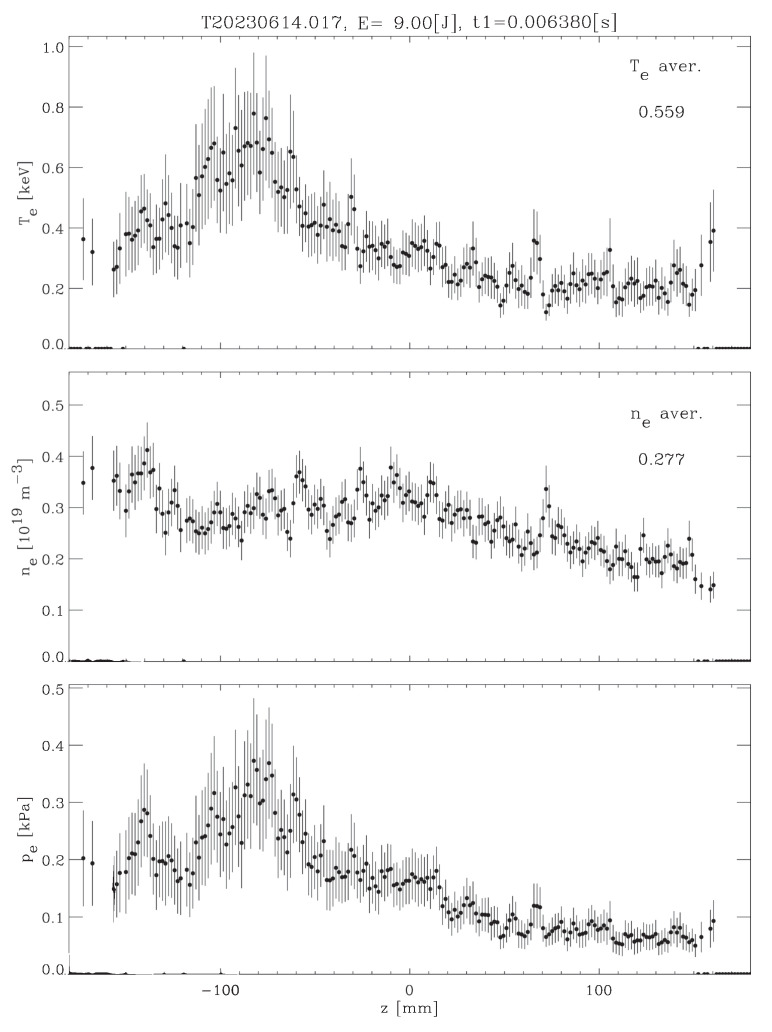
GAN profiles of Thomson scattering diagnosis images.

Figure 11, Figure 12 and Figure 13 show the temperature and density profiles of plasma for the previous original ECH, ERCC ECH, and GAN ECH images. As can be seen, both approaches seem to have similar performance in the stray light reduction process. So to get a better comparison, we used the denoising function D(I) described in Section 4.

Table 2 shows the noise reduction results as a percentage for both algorithms applied to the TS images. The rows represent the algorithms, and the columns correspond to the images. The percentage means the fraction of images without noise, i.e., 100% means that all images for a given ST image type are without noise according to the denoising function (see Equation (1)). While both algorithms demonstrate satisfactory outcomes, it is evident that the GAN algorithm yields superior results.

**Figure 14:**
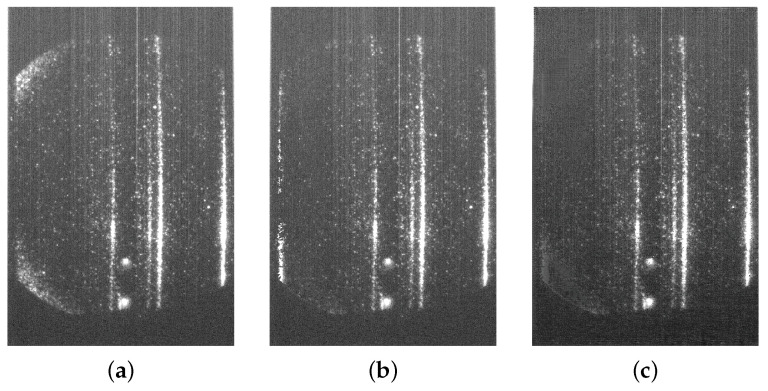
Example of noise duplication: (**a**) Original STR, (**b**) ERCC STR, and (**c**) GAN STR.

Despite its good performance, the ERCC approach has an important drawback. Figure 14 depicts how the algorithm, in some images, duplicates the noise presented on the right side of the image. This is because the algorithm essentially replaces the pixels that have been identified as noise with the pixels on the right-hand side of the image. In contrast, the GAN algorithm manages to eliminate noise from the image without making any duplication.

**Figure 15:**
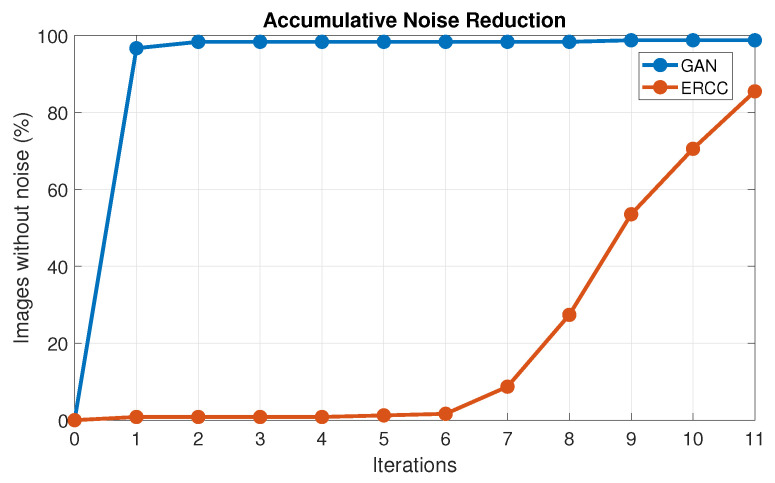
Comparison between both algorithms.

Finally, Figure 15 shows a comparison between both algorithms, taking into account the number of iterations. The y axis represents the percentage of noise reduction, and the x axis represents the number of iterations. Note that in the GAN algorithm, most images have been effectively processed within the first 3 iterations, while the ERCC takes 11 iterations to reach its maximum value. This demonstrates that the GAN algorithm is much faster than the ERCC algorithm, with a direct impact on the time needed to obtain a single image. Specifically, with the ERCC algorithm, it takes around 1.20 s, although this value may vary for different categories of images. On the other hand, the noise-free image generated by the GAN takes 0.20 s, meaning that the time to obtain the data is five times faster for the GAN algorithm.

The GAN model was trained on a server with a Xeon, from Santa Clara, California, USA, scalable processor with 2×10 cores at 2.20 GHz, 256 GB of RAM memory, and 8 GPUs NVIDIA RTX 2080 Ti. On average, the model took one week to be trained. For the testing and validation of the model, a laptop was used. It has an Intel core i7 processor at 2.6 GHz and 16 GB of RAM, and one Nvidia Geforce RTX 2060 graphics card, from Santa Clara, California, USA, was used.

## 6. Conclusions

In the Thomson scattering diagnosis in nuclear fusion, stray light adds disturbance to the measurements and must be reduced/eliminated. To achieve this, an application based on generative adversarial networks (GANs) was designed and used. This network allowed for the “translation” of an image with noise into one without noise, based on unsupervised training. Once the model was tested and validated, the results proved to be reliable, and the model demonstrated the capability to generate data highly similar to those obtained in the TS diagnostic system. To establish reliability, the obtained results were compared with those of the ERCC algorithm. Among the advantages of the proposed method, it stands out for its straightforward implementation, requiring only a good database to obtain a functional model. Additionally, a notable advantage is the significant reduction in processing time to obtain noise-free images. This efficiency results from both the low number of iterations required by the algorithm to produce a clean image and the generative nature of the process, contrasting with the ERCC approach, which inspects images at each iteration. Connected to this, the validation process for noise reduction reveals a substantial increase in the percentage of noise-free images. Another highly relevant aspect of the proposed approach is that the GAN never duplicates information from the right side of the images to the left side, resulting in cleaner images compared to the ERCC algorithm. This phenomenon is reflected in a precise Thomson scattering diagnosis, where preserving image details is essential. Last but not least, the developed method enables the generation of realistic and processed data without the need to modify the original data. In future work, the focus will be on enhancing the reliability of the generated data, a critical point to consider. The developed approach allows for the application of different types of data, thus generating realistic synthetic data that can balance various databases.

## Figures and Tables

**Table 1:** Description of the five types of TJ-II images.

TS Image	Description
BKG	CCD camera background
COFF	Cut-off density during electron cyclotron resonant heating
ECH	Electron cyclotron resonant heating
NBI	Neutral beam injection heating
STR	Stray light

**Table 2:** Comparison of the noise reduction for the approaches (GAN) and the ERCC algorithms.

	BKG	COFF	ECR	NBI	STR	Total
GAN	100%	100%	100%	100%	94%	99%
ERCC	74%	100%	94%	94%	65%	85%

## Data Availability

Data are contained within the article.

## References

[1] Barbarino M. (2022). On the brink of a new era in nuclear fusion R&D. Nat. Rev. Phys..

[2] Mathew M. (2022). Nuclear energy: A pathway towards mitigation of global warming. Prog. Nucl. Energy.

[3] Murph S.E., Murph M.A. (2022). Nuclear fusion: The promise of endless energy. Phys. Sci. Rev..

[4] Vega J., Murari A., Dormido-Canto S., Rattá G., Gelfusa M., JET Contributors (2022). Disruption prediction with artificial intelligence techniques in tokamak plasmas. Nat. Phys..

[5] Ishida S., Barabaschi P., Kamada Y., JT-60SA Team (2011). Overview of the JT-60SA project. Nucl. Fusion.

[6] Glugla M., Lässer R., Dörr L., Murdoch D., Haange R., Yoshida H. (2003). The inner deuterium/tritium fuel cycle of ITER. Fusion Eng. Des..

[7] Hirsch R., Bezdek R. (2021). Public Acceptance of ITER-Tokamak Fusion Power. Eur. J. Energy Res..

[8] Stork D. (2009). DEMO and the Route to Fusion Energy.

[9] Farias G., Correa R., Fabregas E., Dormido-Canto S., Vega J., Pastor I. Deep learning models to generate realistic new data in nuclear fusion. Proceedings of the 13th Technical Meeting on Plasma Control Systems, Data Management and Remote Experiments in Fusion Research, IAEA.

[10] Farias G., Dormido-Canto S., Vega J., Martínez I., Hermosilla G., Fabregas E. (2018). Image classification by using a reduced set of features in the TJ-II Thomson scattering diagnostic. Fusion Eng. Des..

[11] Farias G., Fabregas E., Dormido-Canto S., Vega J., Vergara S., Dormido S., Pastor I., Olmedo A. (2018). Applying deep learning for improving image classification in nuclear fusion devices. IEEE Access.

[12] Farias G., Dormido-Canto S., Vega J., Pastor I., Santos M. (2013). Application and validation of image processing algorithms to reduce the stray light on the TJ-II Thomson scattering diagnostic. Fusion Sci. Technol..

[13] Farias G., Dormido-Canto S., Vega J., Santos M., Pastor I., Fingerhuth S., Ascencio J. (2014). Iterative noise removal from temperature and density profiles in the TJ-II Thomson scattering. Fusion Eng. Des..

[14] Pearson R., Antoniazzi A., Nuttall W. (2018). Tritium supply and use: A key issue for the development of nuclear fusion energy. Fusion Eng. Des..

[15] Boozer A. (2021). Stellarators as a fast path to fusion. Nucl. Fusion.

[16] Juarez R., Pedroche G., Loughlin M., Pampin R., Martinez P., De Pietri M., Alguacil J., Ogando F., Sauvan P., Lopez-Revelles A. (2021). A full and heterogeneous model of the ITER tokamak for comprehensive nuclear analyses. Nat. Energy.

[17] Knapp V., Pevec D. (2018). Promises and limitations of nuclear fission energy in combating climate change. Energy Policy.

[18] Hora H., Fuerbach A., Ladouceur F., McKenzie W. (2021). Green energy generation via optical laser pressure initiated nonthermal nuclear fusion. Opt. Eng..

[19] Rebut P., Bickerton R., Keen B. (1985). The Joint European Torus: Installation, first results and prospects. Nucl. Fusion.

[20] Boedo J., Rudakov D., Moyer R., McKee G., Colchin R., Schaffer M., Stangeby P., West W., Allen S., Evans T.E. (2003). Transport by intermittency in the boundary of the DIII-D tokamak. Phys. Plasmas.

[21] Alejaldre C., Alonso J., Almoguera L., Ascasíbar E., Baciero A., Balbín R., Blaumoser M., Botija J., Brañas B., De la Cal E. (1999). First plasmas in the TJ-II flexible heliac. Plasma Phys. Control. Fusion.

[22] National Fusion Laboratory of Spain (CIEMAT) (2008). TJ-II Project.

[23] Dinklage A., Beidler C., Helander P., Fuchert G., Maaßberg H., Rahbarnia K., Sunn Pedersen T., Turkin Y., Wolf R., Alonso A. (2018). Magnetic configuration effects on the Wendelstein 7-X stellarator. Nat. Phys..

[24] Lee S., Bak J., Ka E., Kim J., Hahn S. (2008). Magnetic diagnostics for the first plasma operation in Korea superconducting tokamak advanced research. Rev. Sci. Instruments.

[25] Streibl B., Lang P.T., Leuterer F., Noterdaeme J.M., Stäbler A. (2003). Chapter 2: Machine design, fueling, and heating in ASDEX Upgrade. Fusion Sci. Technol..

[26] Breault R.P. (1995). Control of Stray Light. Handbook of Optics.

[27] de Sande M.V. (2002). Laser Scattering on Low Temperature Plasmas. Ph.D. Thesis.

[28] Herranz J., Pastor I. Influence of the stray light on the recorded Thomson electronic distribution function. Proceedings of the 15th International Stellarator Workshop.

[29] Foster D. (2019). Generative Deep Learning: Teaching Machines to Paint, Write, Compose, and Play.

[30] Bok V., Langr J. (2019). GANs in Action: Deep Learning with Generative Adversarial Networks.

